# Comparative Proteomic Study Shows the Expression of Hint-1 in Pituitary Adenomas

**DOI:** 10.3390/diagnostics11020330

**Published:** 2021-02-17

**Authors:** Carolina Carrillo-Najar, Daniel Rembao-Bojórquez, Martha L. Tena-Suck, Sergio Zavala-Vega, Noemí Gelista-Herrera, Miguel A. Ramos-Peek, Juan L. Gómez-Amador, Febe Cazares-Raga, Fidel de la Cruz Hernández-Hernández, Alma Ortiz-Plata

**Affiliations:** 1 Experimental Neuropathology Laboratory, National Institute of Neurology and Neurosurgery “Manuel Velasco Suárez”, Insurgentes Sur 3877, Mexico City 14269, Mexico; carocn54@gmail.com; 2 Neuropathology Department, National Institute of Neurology and Neurosurgery “Manuel Velasco Suárez”, Insurgentes Sur 3877, Mexico City 14269, Mexico; jdrbojorquez2002@gmail.com (D.R.-B.); mltenasuck@gmail.com (M.L.T.-S.); sergio.zavala.vega@gmail.com (S.Z.-V.); ngelistaherrera@yahoo.com.mx (N.G.-H.); 3 Neurosurgery Division, National Institute of Neurology and Neurosurgery “Manuel Velasco Suárez”, Insurgentes Sur 3877, Mexico City 14269, Mexico; marpneurocir@hotmail.com (M.A.R.-P.); jlga@neurocirugia-innn.com (J.L.G.-A.); 4 Department of Infectomics and Molecular Pathogenesis, Center for Research and Advanced Studies of National Polytechnic Institute, IPN Avenue 2508, Mexico City 07360, Mexico; fcazares@cinvestav.mx (F.C.-R.); cruzcruz@cinvestav.mx (F.d.l.C.H.-H.)

**Keywords:** pituitary adenomas, neuroendocrine tumors, proteomic analysis, mass spectrometry, invasive, biological behavior, biomarkers, classification

## Abstract

Pituitary adenomas (PAs) can be unpredictable and aggressive tumors. No reliable markers of their biological behavior have been found. Here, a proteomic analysis was applied to identify proteins in the expression profile between invasive and non-invasive PAs to search for possible biomarkers. A histopathological and immunohistochemical (adenohypophyseal hormones, Ki-67, p53, CD34, VEGF, Flk1 antibodies) analysis was done; a proteomic map was evaluated in 64 out of 128 tumors. There were 107 (84%) invasive and 21 (16%) non-invasive PAs; 80.5% belonged to III and IV grades of the Hardy–Vezina classification. Invasive PAs (*n* = 56) showed 105 ± 43 spots; 86 ± 32 spots in non-invasive PAs (*n* = 8) were observed. The 13 most prominent spots were selected and 11 proteins related to neoplastic process in different types of tumors were identified. Hint1 (Histidine triad nucleotide-binding protein 1) high expression in invasive PA was found (11.8 ± 1.4, *p* = 0.005), especially at high index (>10; *p* = 0.0002). High Hint1 expression was found in invasive VEGF positive PA (13.8 ± 2.3, *p* = 0.005) and in Flk1 positive PA (14.04 ± 2.28, *p* = 0.006). Hint1 is related to human tumorigenesis by its interaction with signaling pathways and transcription factors. It could be related to invasive behavior in PAs. This is the first report on Hint expression in PAs. More analysis is needed to find out the possible role of Hint in these tumors.

## 1. Introduction

Pituitary adenomas (PAs) are the most common alteration of the pituitary gland. At the National Institute of Neurology and Neurosurgery in Mexico City, they are the second most treated tumor after meningeal tumor (22.35%) [1] and account for 91% of sellar and parasellar tumors and 10–15% of intracranial neoplasms with prevalence 2.7–24% in autopsy investigations [2,3,4]. They have monoclonal origin and are classified according to their hormonal type (lactotrophs, gonadotrophs, somatotrophs, corticotrophs, and thyrotrophs). Prolactinomas and adrenocorticotropic hormone (ACTH) PAs are the most common in women, while non-functioning pituitary adenomas (NFPAs) and growth hormone PAs are the most frequent in men [5,6,7,8]. They are classified into functioning or non-functioning pituitary adenomas according to their clinical manifestations related or unrelated to hormone production, and by their size evaluated with imaging studies (microadenomas: ≤10 mm diameter; macroadenomas: ≥10 mm diameter; or giant adenomas: ≥40 mm diameter) [9,10]. They have been considered benign and slow-growing tumors with complex and varied clinical manifestations in relation to hormonal mismatch (galactorrhea, amenorrhea, Cushing’s syndrome, acromegaly, gigantism) or mass effect (headache, visual impairment). Despite their apparent behavior, PAs can cause considerable damage with significant morbidity (diabetes: up to 80%; hypertension: 50%; visual defect: 30.5%; neurosurgery effects: 86.1%), and mortality (up to 16%) [11,12,13]. They can be invasive (30–45% of the cases), destroying adjacent structures as sphenoid and/or cavernous sinus and bones at the base of the skull (grades III and IV of Hardy–Vezina classification system) and invading the cavernous sinus (grades III and IV of Knosp classification system) [14,15,16]. In addition, PAs can be aggressive, presenting rapid growth, significant invasion, resistance to conventional treatment, recurrences (sometimes needing more than one surgery in a period of seven years), increased mitotic index, Ki67 proliferation index (Ki67i) greater than 3%, and extensive p53 immunostaining. These tumors were considered a high risk and were classified as atypical (third edition of WHO classification), but the atypical pituitary term is no longer used in the new WHO classification (2017) [17,18]. Due to the aggressive and invasive character of PAs, the term neuroendocrine pituitary tumor (PitNet, pituitary neuroendocrine tumor) has been proposed, which better describes their behavior [15].

In PA investigation, several experimental strategies (histochemistry and immunohistochemistry techniques) have been performed, which allow for the identification and classification of PAs according to stain affinity (basophils, chromophobes, and acidophils), type of hormonal content (prolactin, growth hormone, LH, FSH, TSH, ACTH), PCR technique (endpoint PCR, RT-PCR, ddPCR), microarrays, RNA-Seq, bidimensional polyacrylamide gel electrophoresis (2-DE), mass spectrometry, and laser-capture microdissection (LCM) [19,20,21,22,23,24,25,26]. Furthermore, DNA sequencing has been applied to find genetic alterations and epigenetic changes [27], but only partial tumor genesis information has been found, since the transcriptome and the proteome are dynamic. Then, new experimental strategies have been used in an effort to elucidate the biological behavior of PAs. The term proteome was first introduced by Wasinger and Wilkins to define the analysis of gene expression based on the analysis of proteins, where the proteome is the PROTEin analysis, the complement expressed by the genOME of a cell or tissue [28,29]. This is why transcriptomics and proteomics play a key role in functional genomics to understand the regulation of biological systems.

The proteomic analysis of the PA has revealed the expression of proteins classified by functional groups (pituitary hormones, cellular signals, enzymes, cellular-defense proteins, and cell-structure proteins, among others) among different subtypes of tumors. They are related to pathway networks (Wnt and Notch pathways; mitochondrial dysfunction, oxidative stress, MAP kinase, PI3K-Akt, mTOR ERK/MAPK, endocytosis, and spliceosome signaling pathways, among others), some of them associated with tumor invasiveness and aggressiveness [30,31,32,33,34,35,36,37]. Variants of prolactin have been identified in prolactinomas, and they may be involved in different signaling pathways [38]. In ACTH-secreting PAs, differentially expressed proteins have been found to be related to the Myc signaling pathway and participate in metabolic changes and tumorigenesis in these tumors [39]. Differentially expressed proteins, genes, and mRNA isoforms have been identified through integrative proteomics and transcriptomics; they have been proposed as markers of invasiveness and possible therapeutic targets [40,41,42,43]. However, further research is necessary to consider some proteins as diagnosis, prognosis, and/or treatment biomarkers.

The aim of this study was to analyze proteomic profiles in invasive and non-invasive PAs to search for proteins associated with the pathophysiology of these tumors. We analyzed proteomic profiles from PA tissues by 2DE, protein spots were selected and identified by mass spectrometry, and Hint1 protein expression was assessed in PA. The results of this work show the search for proteins participating in the neoplastic process that are possible candidates for diagnostic, prognostic, and/or treatment markers.

## 2. Materials and Methods

The project was approved by the ethics committee of the National Institute of Neurology and Neurosurgery (101-17), and the study was conducted in accordance with the Declaration of Helsinki. Informed consent for the use of the samples for research purposes was obtained from the patients.

**Figure 1:**
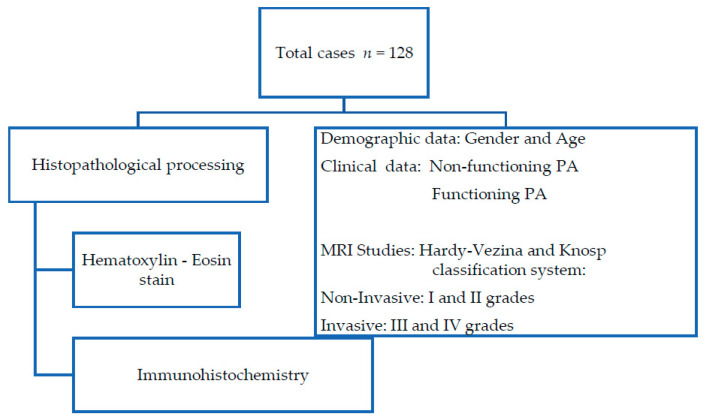
Flowchart of histopathological procedure and clinical data obtained from medical records.

A total of 128 PA samples were collected and processed for histopathological analysis. The PAs were classified according to their clinical manifestations and radiological (MRI) and histopathological analysis (Figure 1).

### 2.1. Histopathological Processing

Surgical specimens for the patient’s diagnosis of PAs were collected during surgery. The surgical procedure performed for tumor resection was transnasal–transsphenoidal and transcranial. PA tissue intended for histopathological analysis was fixed with 10% formaldehyde, dehydrated in an automatic tissue processor using alcohol (Histokinette 2000, Reichert-Jung, American Optical Buffalo, New York, NY, USA), and embedded in paraffin. Four micrometer-thick sections were stained with hematoxylin–eosin and observed under a wide-field Nikon photo microscope (Nikon Co., Tokyo, Japan). Neuropathologist experts analyzed the biopsies and determined the histopathological diagnostic. PA were characterized by immunohistochemistry with adenohypophyseal hormones, proliferation, and angiogenic markers.

### 2.2. Immunohistochemistry

Sections of each case were deparaffinized, rehydrated, and rinsed in phosphate-buffer saline (PBS); antigenic retrieval was performed heating the sections in a pressure cooker in a commercial solution (Reveal, Biocare Medical, Concord, CA, USA) for 7 min, and rinsed again in PBS. Afterwards, endogenous peroxidase was blocked with 0.25% H_2_O_2_/distilled water for 20 min, rinsed in PBS at first, and then in 0.1%-Triton X-100 PBS. The sections were incubated in a wet chamber, in primary antibodies against adenohypophyseal hormone antibodies (Prl, GH, LH, FSH, ACTH, TSH), proliferation marker antibodies (Ki67, p53), angiogenic marker antibodies (CD34, VEGF, Flk1), and Hint1 protein (Table 1) at 4 °C overnight. Slides were washed after incubation with the primary antibody, and the reaction was detected by streptavidin–biotin system with the Peroxidase Mouse & Rabbit kit (Diagnostic BioSystems, Pleasanton, CA, USA) and revealed with diaminobenzidine using a 2 Component DAB Pack kit (BioGenex, Carpinteria, CA, USA) according to the manufacturer’s instructions; by the last, sections were hematoxylin counterstained. Normal human pituitary tissue sections were used as a positive control of the immunohistochemical technique, and sections incubated without primary antibody were used as negative control. Immunodetection was analyzed by two experts, under a wide-field photo microscope Nikon (Nikon Co. Tokyo, Japan).

The assessment of immunohistochemical reaction (adenohypophyseal hormones, p53, VEGF, Flk1) was reported as positive in tissues with constant brown stain (nuclear or cytoplasmic) throughout the histological section, ruling out a scant or sporadic mark. Ki67i and Hint1 proteins were evaluated by quantifying the number of positively stained nuclei in five to 10 high power fields at ×400 per case and were reported as mean value of positive nuclei. For the evaluation of microvascular density (CD34d), blood positive vessels were quantified in the three most vascularized areas (hot spots) of the tumors at ×400 magnification (×40 objective lens) per case, and the average of each slide was obtained [44].

All data were presented as mean ± SD. To analyze the variables, the Kolmogorov–Smirnov test was performed, and the data were analyzed by one-way ANOVA (IBM SPSS Statistics v. 25.0; GraphPad Prism 5). To identify whether gender, age, MRI characteristics, or hormone expression on tissue were associated with the expression of Ki-67i, p53, CD34, VEGF, Flk1, and Hint1, Student’s *t*-test, analysis of variance, and chi-square test were used; *p* < 0.05 was considered statistically significant.

### 2.3. Proteomic Process

#### 2.3.1. Protein Pituitary Adenoma Extraction

During neurosurgery, a dry tumor sample was collected in a sterile Eppendorf tube and kept on ice immediately to avoid degradation. The sample was evaluated by a neuropathologist to separate tumor tissue and stored at −80 °C until process. PA proteins were analyzed by 2-DE using immobilized pH gradient strips [45]. Tumor tissue samples were homogenized in lysis buffer (7 M urea, 2 M thiourea, 4% CHAPS, 2% IPG buffer pH 3–10 (GE, Healthcare, Piscataway, NJ, USA), 40 mM DTT) with protease inhibitor cocktail (Complete, Roche Diagnostics, Indianapolis, IN, USA) and phosphatase inhibitors (PhosStop, Roche Diagnostics, Penzberg, Germany) followed by three freeze/thaw cycles in liquid nitrogen. Then, the samples were centrifuged at 14,000 × g at 4 °C for 30 min, and the supernatant was recovered and subsequently precipitated with acetone at −20 °C with methanol/ chloroform. Finally, the proteins were cleaned with a 2-D Clean-Up kit (GE Healthcare, Piscataway, NJ, USA) according to the manufacturer’s instructions. The samples were solubilized in rehydration buffer (Destreak Rehydration Solution, GE Healthcare, USA) with protease and phosphatase inhibitors. Protein quantification was done using a 2-D Quant Kit (GE Healthcare, Biosciences, USA) according to the supplier’s instructions. One hundred fifty μL samples (5.153 μg/10 μL ± 1.784; 77 ± 26.8 μg protein) were applied to IPG strips pH 3–10, 7 cm (GE Healthcare, Sweden), and rehydrated at room temperature for 16 h. Afterwards, IEF was carried out in a Protean IEF Cell (Bio-Rad, Hercules, CA, United States), following the manufacturer’s protocol. Second dimension was performed in 15% SDS-PAGE, and gels were stained with Bio-Safe Coomassie (Bio-Rad, USA).

#### 2.3.2. Image and Data Analysis of Gel

Images of 2-DE gels were obtained using the Fusion FX 6 Edge V. 070 imaging system (Vilber Lourmat, Collégien, France), and the proteomic profile of each biopsy was analyzed, using Bio1D EvolutionCapt (Vilber Lourmat, Collégien, France). Protein spots were quantified in each 2-DE gel and compared in invasive and non-invasive PA; differential protein spots (different between invasive and non-invasive PAs) were selected, considering those that showed outstanding intensity and a better definition. The quantification number of 2-DE spots was analyzed by Student’s *t*-test.

#### 2.3.3. Nanoflow LC-MS/MS

Protein identification was done at PlanTECC National Laboratory of the Center for Research and Advanced Studies at the National Polytechnic Institute, Campus Irapuato, Guanajuato Mexico. All experiments were performed on a nanoACQUITY nano-flow liquid chromatography (LC) system, coupled to an LTQ velos linear ion trap mass spectrometer (Waters, Thermo Fisher Scientific, Bremen, Germany) equipped with a nanoelectrospray ion source.

The selected spots were manually excised from Coomassie blue 2-DE gels under sterile conditions; proteins were extracted and trypsin was digested [46]. Immediately afterwards, 3 µl digested proteins were resuspended in solvent A (0.1% formic acid) and bound to a pre-column (Symmetry^®^ C18, 5 μm, 180μm × 20 mm, Waters). Subsequently, the flow was then switched to a 10-cm capillary UPLC column (100 μm ID BEH-C18 1.7μm particle size). The column temperature was controlled at 35 °C. The peptides were separated by a 60-min gradient method at a flow rate of 400 nL/min. The gradient was programmed as follows: 3–50% solvent B (100% acetonitrile in 0.1% formic acid) over 30 min, 50–85% B over 2 min, 85% B over 4 min, and 3% B over 22 min. The peptides were eluted into the mass spectrometer nano-electrospray ionization source through a standard coated silica tip (NewObjective, Woburn, MA, USA). The mass spectrometer was operated in data-dependent acquisition mode in order to automatically alternate between full scan (400–1600m/z) and subsequent Top 5 MS/MS scans on the linear ion trap. Collision-induced dissociation was performed using helium as collision gas at a normalized collision energy of 35% and 10 ms activation time. Data acquisition was controlled using Xcalibur v2.3 (Thermo Fisher Scientific).

#### 2.3.4. Automated Data Evaluation Work-Flow

Tandem mass spectra were extracted in Proteome Discoverer v1.4 and searched against a database on a Sequest HT engine. Searches were executed with the following parameters: 2 Da parent MS ion window, 1 Da MS/MS ion window, and two missed cleavages allowed. The iodoacetamide derivative of cysteine (carbamidomethylcysteine) was specified on Sequest as a fixed modification and oxidation of methionine as a variable modification.

## 3. Results

### 3.1. Clinical Characteristics

Demographic and clinical dates of PA are presented in Table 2.

Recurrences have no relation with gender (*p* = 0.437), hormonal PA types (*p* = 0.116), or between invasive and non-invasive PAs (*p* = 0.983). These results are in accord with those previously reported. Although PA invasiveness has been associated with recurrence, studies have concluded that the invasive behavior itself is not a significant factor in predicting recurrence. Furthermore, suprasellar extension and/or cavernous sinus invasion are also not associated with tumor recurrence and, although larger tumors are found to recur more frequently, no statistically significant differences were found. [47].

### 3.2. Histopathological Findings

**Figure 2:**
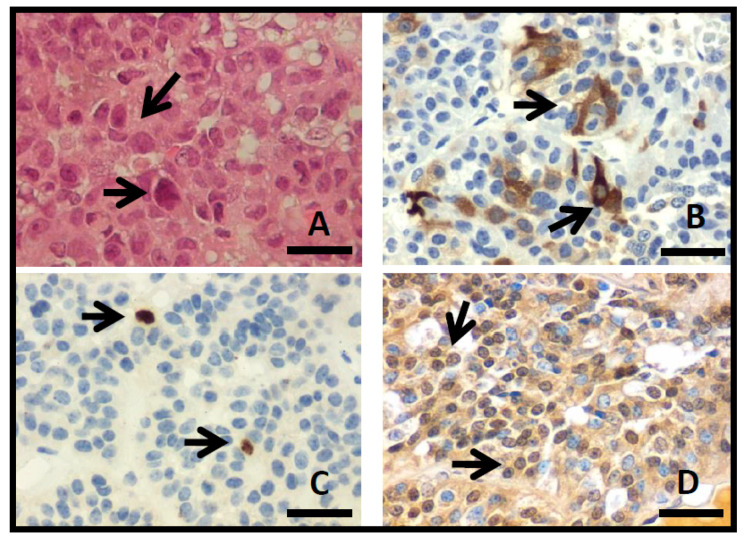
(**A**) Photomicrographs of PA with hematoxylin–eosin stain, showing solid pattern with nuclear pleomorfism (arrows). Immunohistochemistry show cytoplasmic detection to FSH (**B**), Ki-67 nuclear detection (**C**), and p53 nuclear detection (**D**) (arrows; original magnification 400×, scale bar 250 µm).

The tissue sections showed an epithelial neoplasm with solid, papillary, and nodular histological patterns. Scarce biopsies with cellular atypia (7%), nuclear pleomorphism (1.6%), and mitosis (0.8%) were observed (Figure 2A). According to the immunohistochemistry analysis, 70 (54.7%) were FSH and/or LH-positive PA, 26 (20.3%) showed plurihormonal content, 20 (15.6%) exhibited no hormonal detection, four (3.1%) were GH-PA, three (2.3%) presented prolactin hormone, four (3.1%) were ACTH, and one (0.8%) was prolactin-GH (Figure 2B). Cell proliferation was assessed by immunohistochemistry, and Ki-67i expression (Figure 2C) was 0.7 ± 0.08% (range, 0–3.6%). No significant difference was found between invasive (range, 0–3.6; 0.8 ± 0.08) and non-invasive PAs (range, 0–3.2; 0.6 ± 0.2) (*p* = 0.341). The expression of p53 showed 97 (75.8%) positive cases (Figure 2D), out of which 82 (84.5%) were invasive Pas, but no statistically significant difference was observed between invasive and non-invasive PAs (*p* = 0.611). No relation between the positive expression of p53 and Ki-67i was found (*p* = 0.919).

**Figure 3:**
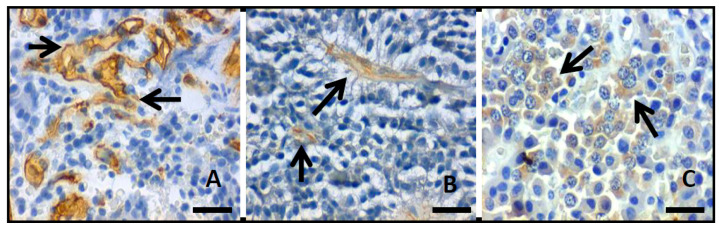
Photomicrographs showing the inmmunohistochemistry reaction (arrows) to (**A**) CD34, (**B**) VEGF, and (**C**) Flk1.; original magnification 400×, scale bar 250µm).

Angiogenic grade in solid tumors, as PA, is commonly evaluated by microvascular density in which the number of vessels in a certain area are quantified. The CD34d (Figure 3A) was 5.1 ± 3.9 (range, 1–33.3), while no statistical difference was found between invasive (CD34d 5.3 ± 3.1) and non-invasive PAs (CD34d 6.3 ± 6.6) (*p* = 0.274). VEGF expression was found in 49 (38.3%) cases (Figure 3B), out of which 41 (83.7%) were invasive PA; still no statistically significant difference was found between invasive and non-invasive PAs (*p* = 0.563). The expression of Flk1 was positive in 36 (28.1%) PAs, out of which 33 (91.7%) were invasive (Figure 3C). There was no statistically significant difference between invasive and non-invasive PAs (*p* = 0.147). An inverse relationship was found between the positive expression of Flk1 and Ki67i (*p* = 0.030) such that a lower Ki67i was observed in positive Flk1 PAs. Additionally, a relation between Flk1 and positive p53 expression was found (*p* = 0.031). No relation was found between recurrence with CD34 (*p* = 0.374), VEGF (*p* = 0.696), and Flk1 (*p* = 0.972).

No statistically significant difference was found between hormonal PA types and Ki-67 (*p* = 0.182), p53 (*p* = 0.733), CD34 (*p* = 0.620), VEGF (*p* = 0.138), Flk1 (*p* = 0.266), and gender (*p* = 0.095).

These results show that even though 80.5% of the cases studied are invasive, they do not show aggressive behavior. It has been shown that the term invasiveness in PA is not synonymous with aggressiveness. Aggressive PAs show a Ki-67 index >3%, extensive p53 positivity, and a high rate of mitosis, recurrence, and resistance to treatment. These factors were not observed in the cases studied; still, it is necessary to follow up these patients, since this was the first surgery for most of them [16,18].

### 3.3. Proteomic Analysis

Demographic and clinical data of PAs analyzed by proteomics are shown in Table 3.

**Figure 4:**
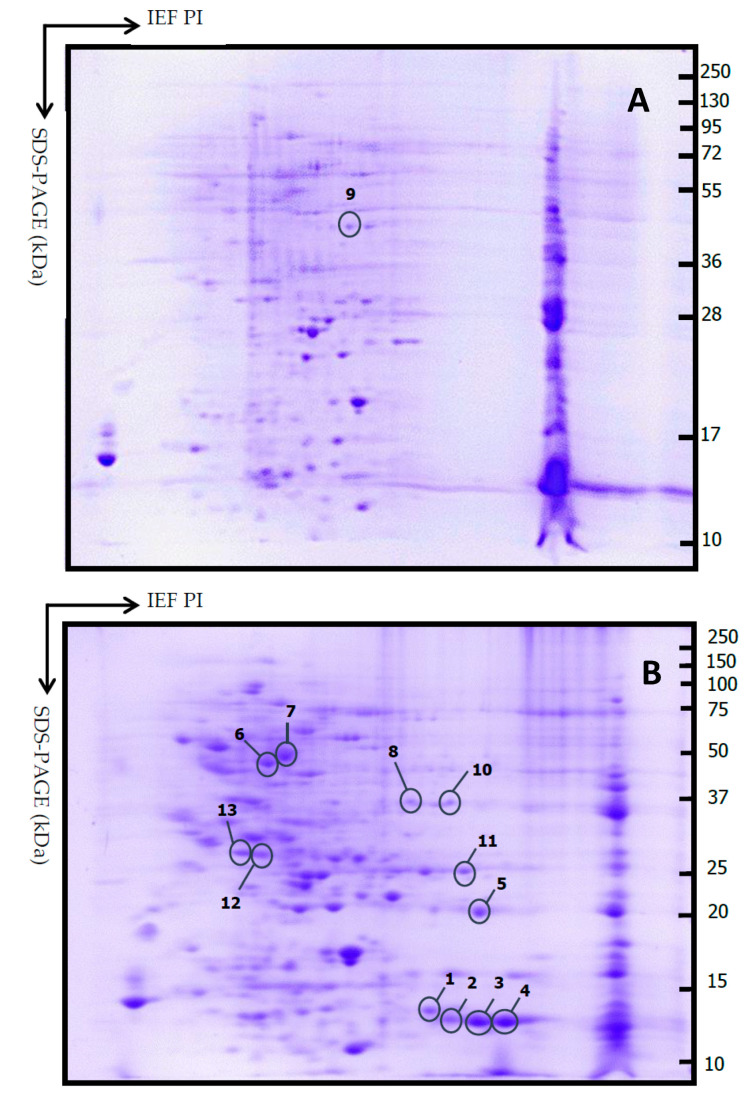
Representative 2DE gels from non-invasive PA (**A**) and invasive PA (**B**). Thirteen spots were selected comparing between all gels. Isoelectric focusing (IEF) was carried out with a 7 cm IPG strip (pH 3–10); second dimension SDS-PAGE (15% polyacrylamide gel).

Sixty four samples out of 128 cases were collected and analyzed by 2-DE. The availability of tissue for proteomic analysis depended on the amount of tumor tissue obtained during surgery. The tissue was divided into two parts: one sample was used in histopathology to make the diagnosis and the other in proteomic analysis. In the proteomic analysis, 103.4 ± 42.3 spots (range, 32–260) were found distributed in the area of pH 4.0–8.0 and mass 10–100 kDa. Invasive PAs (*n* = 56) showed 105 ± 43 spots, while 86 ± 32 spots were observed in non-invasive PA (*n* = 8). No statistical difference was found between invasive and non-invasive PAs (*p* = 0.226). A master 2-DE gel was selected for invasive and non-invasive Pas, and the most representative and differential spots of each group were selected (Figure 4). Thirteen differential spots were selected, and 11 proteins were identified in the proteomic profile (Table 4).

### 3.4. Hint1 Expression

**Figure 5:**
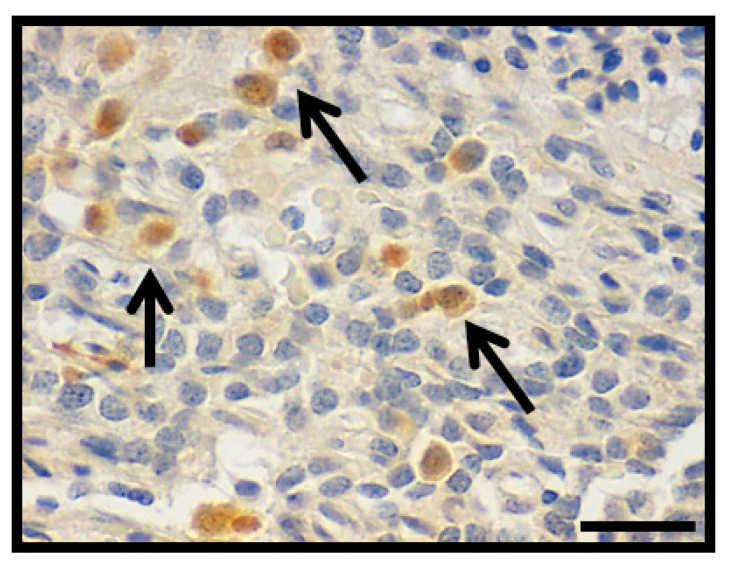
Photomicrograph showing the nuclear inmmunohistochemistry detection to Hint1 in PA (arrows; original magnification 400×, scale bar 250 µm).

In order to rectify the presence of Hint1 in PA, Hint1 expression index (Hint1i) was assessed by immunohistochemistry in the same 64 cases where Hint1 was identified (Figure 5). PAs show Hint1i 10.9 ± 1.3 (range 0.5–38.1). Non-invasive PAs show Hint1i 4.8 ± 0.94 (range 2.6–9); a statistically significant difference was found between non-invasive and invasive PAs (11.8 ± 1.46, *p* = 0.005). Since non-invasive PAs showed a range of expression of 2.6–9, and the invasive PAs showed a wider range of expression, the values were grouped in two levels: low level <10 and high level >10. A statistically significant difference was found between non-invasive and invasive PAs with a high Hint1i (Hint1i >10; *p* = 0.0002). Statistically significant differences were found between Ki-67i in low Hint1i (0.95 ± 0.15) with Ki-67i in high Hint1i (0.47 ± 0.16; *p* = 0.044). Hint1i was compared against p53 expression. No statistically significant differences were found between Hint1i in p53 positive cases (12.2 ± 1.5) and Hint1i in p53 negative cases (6.4 ± 2.5; *p* = 0.08); however, a statistically significant difference was observed in p53 positive cases between low (5.4 ± 0.5) and high Hint1i (20 ± 0.8; *p* = 0.0001).

Expressions of angiogenic factors were assessed. Statistically significant difference of CD34d between Hint1i positive cases (5.7 ± 0.5) and Hint1i negative cases (3.73 ± 0.5; *p* = 0.007) was found. Hint1i showed a statistically significant difference in VEGF positive cases between invasive (13.8 ± 2.3) and non-invasive PA (4.8 ± 0.28; *p* = 0.005), and between Flk1 positive cases (14.04 ± 2.27) and Flk1negative cases (6.5 ± 1.3; *p* = 0.006). No relation was found between Hint1 expression and tumor size (*p* = 0:822), gender (*p* = 0.560), hormonal type (*p* = 0.953), and recurrence (*p* = 0.295).

## 4. Discussion

The pituitary gland is made up of different cell types, each one related (each cell type) to its hormone secretion. Therefore, PAs are a heterogeneous group of tumors due to their monoclonal origin, giving rise to different tumor types. PAs have been classified according to their radiological, clinical, and histopathological characteristics as well as their hormonal content. In this cellular diversity, many molecular processes participate, complicating the study and limiting the understanding of PAs. Despite being considered benign, PAs can be invasive, aggressive, and recurrent, causing deterioration in human health. Markers are necessary to explain their biological behavior and help in their prognosis and treatment. Our study aimed to identify proteins in the expression profile between invasive and non-invasive PAs to search for possible biomarkers. By proteomic strategy, differences in proteomic profile between invasive and non-invasive PAs were observed, and 11 proteins were identified. Proteins related to metabolic enzymes (Phosphoglycerate mutase 1, Gamma enolase), cellular signals (14-3-3 protein epsilon), cell structure and mobility (Tropomyosin alpha 3 chain), and energy metabolism (ATP synthase beta chain, mitochondrial) were found, as reported in PAs [30]. Proteins involved in a neoplastic process were identified, and no reports of their expression in PA were found.

Hint1 (Histidine triad nucleotide-binding protein 1) was first identified as a human PKC-interacting protein whose cDNA encodes a 13.7 KDa protein located in 5q31.2 human chromosome. Later, it was found in normal and tumor cell lines, and its function as PCK inhibitor was discarded [48,71]. Hint1 is a protein member of the histidine triad family (HIT; Hist-X-Hist-X-HistX-X; X = hydrophobic amino acid), and it is a part of a binding loop for the α-phosphate of purine nucleotides. It can bind to nucleotides as AMP, ADP, and diadenosine polyphosphates Ap3A and Ap4A. Rabbit Hint1 can also bind to several purine nucleosides and nucleoside ‘5-phosphates, while rabbit and human Hint1 can hydrolyze ADP in vitro; therefore, Hint1 is considered a purine nucleotide-binding protein. It has been suggested that the biological effects of unusual purine nucleotides can be mediated by Hint1 protein [49,72,73,74,75].

It has been reported that Hint1 can play a role in transcription regulation that could affect tumorigenesis signaling pathways [50], and it has been observed that Hint1 protein can act as tumor-suppressor [48,49]. Hint1 exerts its tumor suppressor activity by binding to transcription factors, such as MITF and β-catenin, and its suppressive function is in turn regulated by an acetylation-dependent mechanism [51,76,77]. Hint1 negative regulation in TCF/β-catenin transcriptional activity was found, which represses the expression of Wnt signaling pathways target genes such as axin2 and cyclinD1 [52]. The expression of SLC20A1 (phosphate transporter 1) in a study on somatotroph adenomas was associated with the activation of the Wnt/β-catenin signaling pathway. In this study, SLC20A1 expression was related to tumor size, invasive behavior, and tumor recurrence [53]. On the other hand, the MITF–Hint1 interaction can be disrupted by the binding of the second messenger Ap4A, and MITF is activated together with post-translational modifications of Hint1 (acetylation and phosphorylation) [78,79]. It has been reported that MITF expression promotes cell proliferation, invasion, and cell survival in rat prolactinomas. MITF can reverse the antitumor effect of miR-137, which has been correlated with invasive behavior in prolactinomas. miR-137 can upregulate Wtn-inhibitory factor1 and inhibit nuclear translocation of β-catenin [78,80].

Cyclin D1 is a cell cycle regulator and can act as oncoprotein. In PAs, the correlation between cyclin D1 expression with Ki67 and tumor size has been reported, and nuclear accumulation of β-catenin and over expression of cyclin D1 and c-Myc was found in non-functioning PA [81,82]. In corticotroph adenomas, cyclin D1 has been proposed as biomarker of tumor aggressiveness [83].

In a transient transfection experiment with Hint1, apoptosis induction associated with high p53 and Bax expression and decreased Bcl-2 expression was observed [84]. The extensive p53 expression and Ki67i >3% have been associated with tumor recurrence in PA [47]. Our analysis found no relation of p53 positive expression to Ki67i; however, high Hint1i (>10) was observed in p53 positive cases and low Ki67i, which could indicate the tumor suppressor activity of Hint1. This result likely shows a possible relationship between the expression of p53 and Ki-67 and Hint1 expression in invasive PA.

The role of Hint1 in cancer migration and invasion has been analyzed. Downregulated Hint1 expression was found in metastatic lymph nodes cells in hepatocellular carcinoma, involving Hint1 in a migration and invasion process by modulating girdin and AKT expression and phosphorylation [54]. Hint1 gene and mRNA expression were assessed in a family history of gastric cancer (FHGC) cases. Higher Hint1 gene expression levels were found in antrum samples with atrophic changes in FHGC cases, while lower mRNA levels were observed in antrum samples of FHGC patients compared against control samples. The decreased Hint1 mRNA levels in FHGC patient samples could be a predisposing marker to develop gastric cancer [85]. We observed high Hint1i expression in invasive PA, and high Ki67i was found in cases of low Hint1i levels. This may point to a necessary closer follow-up of these patients, since the aggressive and invasive behavior of PA can also be related to tumor recurrence [55]. Hint1 has been proposed as potential biomarker of radiosensitivity and therapeutic target. In the gastric cancer cell line SGC-7901, Hint1 inhibits cell proliferation, arrests the cell cycle in G1 phase, and reduces the DNA damage repair induced by radiation, increasing the radiosensitivity [56]. An increase in Hint1 expression by Taraxasterol inhibits the growth of liver cancer cells and regulates Bax, Bcl2, and cyclin D1 expression in human liver cancer [86].

Angiogenesis is an important factor in cell growth, cell differentiation, and endothelial migration and is regulated by vascular endothelial growth factor (VEGF) and its receptor (Flk1). In PAs, VEGF participates in vascular network formation and PA tumorigenesis, as it is involved in cell proliferation and invasion [87,88,89]. In prolactin, PA angiogenesis, together with Ki67 >3%, p53 positive, mitoses >2, and vascular invasion, has been associated with aggressiveness and is suspicious of malignancy. A high rate of VEGF expression has been found in pituitary carcinomas. This factor has been proposed as a marker of poor outcome after partial tumor resection [90,91,92,93]. The VEGF receptor Flk1 (VEGFR-2) is a signal transducer that participates in signaling cascades that can promote endothelial cell survival, proliferation, and angiogenesis, and it has been associated with aggressiveness [87,94]. Although less vascularization has been observed in PA than in a normal pituitary gland, a greater vascular density has been reported in invasive macroprolactinomas [95,96]. Our study found no relation between CD34d and invasive behavior; however, high CD34d was observed in Hint1 positive cases, while Hint1i expression was elevated in invasive VEGF, positive PA, and Flk1-positive cases. Although a relation between Flk1 expression and p53 positive expression was found, an inverse relationship between the Flk1 positive expression and Ki-67i was observed. No reports of Hint1 participation were found in angiogenesis nor vascularization processes. The results of this analysis suggest the possible participation of HINT1 in some signaling pathway that can be related to angiogenesis mechanism. In our work, significant differences were found between invasive and non-invasive PAs. More research is necessary in order to include a greater number of non-invasive PA cases, which is a limitation in this type of study.

## 5. Conclusions

Protein expression analysis by proteomic strategy shows a reference map to assess the cell physiology under a special condition. The heterogeneous nature of pituitary adenomas, given that they are originated by different types of cells, has made it difficult to understand their behavior. Then, it has been necessary to identify biomarkers, tools that allow investigating new pathways involved in their development. Here, we identified the expression of Hint1 protein as related to human tumorigenesis by its interaction with signaling pathways and transcription factors. We found that Hint1 expression is higher in invasive pituitary adenomas, showing a possible relation with the expression of cell proliferation markers and angiogenic factors; it could also be related to invasive behavior. This is a first report on Hint1 expression in pituitary adenomas. Further and more detailed analyses are necessary to understand the signaling pathways in which Hint1 participates and elucidate the possible role of Hint1 in these tumors.

## Figures and Tables

**Table 1:** Antibodies used in immunohistochemical technique.

Antibody	Dilution	Company	Catalog Number
Prolactin	Ready to use	Thermo Scientific	MS-9083-R7
Growth Hormone	Ready to use	BioGenex	AM028-5M
Follicle-stimulating hormone	Ready to use	BioGenex	AM026-5M
Luteinizing hormone	Ready to use	Thermo Scientific	MS-1448-R7
Thyroid stimulating hormone	Ready to use	Thermo Scientific	MS-1453-R7
Adrenocorticotropic hormone	1:50	Dako	M3501
Ki67 (MIB-1)	1:50	Dako	M7240
P53	Ready to use	BioGenex	AM195-5M
CD 34	1:50	Biocare medical	CM084B
VEGF	1:100	Biocare medical	CME356B
Flk-1	1:100	Santa Cruz Biotechnology	Sc-6251
Hint1	1:300	Abcam	124912

**Table 2:** Pituitary adenoma dates.

Dates.	*n*	Frequency %
Cases	128	100
Male	63	49.2
Female	65	50.8
Age (range)	48 ± 12.9 (16–80)	
Size:		
Macroadenoma	111	86.7
Giant	17	13.2
Invasiveness:		
Invasive	107	83.5
Non-functioning	81	75.7
Functioning	26	24.2
Non-invasive	21	16.4
Non-functioning	13	61.9
Functioning	8	38.1
Radiological Classificación		
Hardy–Vezina	*n* = 114	
I	2	1.7
II	21	18.4
III	50	43.8
IV	41	35.9
Knosp	*n* = 48	
I	5	10.4
II	11	22.9
III	16	33.3
IV	16	33.3
Recurrence (number of neurosurgery):		
1	73	76.8
2	12	12.6
3	7	7.3
4	2	2.1
5	1	1.1
Surgical procedure		
transnasal–transsphenoidal	106	82.8
Transcranial	22	17.2

**Table 3:** Pituitary adenoma data.

Date	*n*	Frequency %
Patients with dry sample	64	100
Male	30	46.8
Female	34	53.1
Age (range)	48 years (19–76)	
Tumor size:		
Macroadenoma	54	84.3
Giant adenoma	10	15.6
Invasiveness:		
Invasive	56	87.5
Non-functioning	49	87.5
Functioning	7	12.5
Non-invasive	8	12.5
Non-functioning	4	50
Functioning	4	50
Radiological classification:		
Hardy–Vezina		
I	1	1.7
II	6	10.3
III	29	50
IV	22	37.9
Knosp		
I	5	8.6
II	5	21.7
III	9	39.1
IV	7	30.4
Recurrence (surgery number):		
1	53	82.8
2	4	6.2
3	6	9.3
4	0	0
5	1	1.5

**Table 4:** Mass-spectrometric identification from the selected spots.

Spot	Accession	Protein Name	Score	Coverage	MW (KDa)	pI	Description	Ref
1	P49773	Histidine triad nucleotide-binding protein 1 OS = Homo sapiens OX = 9606 GN = HINT1 PE = 1 SV = 2–(HINT1_HUMAN)	290.95	85.71	13.8	6.95	Tumoral suppressor	[48,49,50,51,52,53,54,55,56]
2	O60739	Eukaryotic translation initiation factor 1b OS = Homo sapiens OX = 9606 GN = EIF1B PE = 1 SV = 2–(EIF1B_HUMAN)	67.60	66.37	12.8	7.37	Related with translation regulation, cell growth, and oncogenesis	[57,58]
3	P04080	Cystatin-B OS = Homo sapiens OX = 9606 GN = CSTB PE = 1 SV = 2–(CYTB_HUMAN)	61.87	45.92	11.1	7.56	Implicated in various cancer types (lung, colon, liver, ovarian, gastric, breast); proposed as potential prognostic marker.	[59,60]
5	NP_002558.1	Phosphatidylethanolamine-binding protein 1 (Homo sapiens)	1766.8	91.98	21	7.53	Involved in various types of cancer. Could act as a metastasis suppressor gene.	[61]
5	NP_001008274.1	Transgelin-3 (Homo sapiens)	268.99	76.38	22.5	7.33	Expressed in tumors with aggressive behavior; related to poor prognosis. Possible tumor suppressor.	[62,63]
6	NP_001966.1	Gamma-enolase (Homo sapiens)	4624.29	90.09	47.2	5.03	Metabolic enzyme Tumoral marker	[30]
7	NP_001677.2	ATP synthase subunit beta, mitochondrial precursor (Homo sapiens)	5822.52	79.40	56.5	5.40	Energy metabolism Found in non-small cell lung cancer; colon, breast, and prostate cancer	[30,64,65]
9	NP_000691.1	Annexin A1 (Homo sapiens)	804.87	76.59	38.7	7.02	Expression contributes to the development and progression of cancer	[66]
11	NP_002620.1	Phosphoglycerate mutase 1 isoform 1 (Homo sapiens)	314.94	80.71	28.8	7.18	Metabolic enzyme Related with cell proliferation, migration, invasion, and apoptosis. In renal, hepatocellular, lung, breast, and colorectal cancer.	[30,67]
12	XP_005256841.1	14-3-3 protein épsilon isoform X1 (Homo sapiens)	302.27	61.67	27.4	4.89	Cell signaling in apoptosis, mitosis, and cell cycle. Found in small cell lung cancer, squamous cell laryngeal, and renal carcinoma and central nervous system tumors.	[30,68,69]
13	NP_001036816.1	Tropomyosin alpha-3 chain isoform Tpm3.2cy (Homo sapiens) charged multivesicular body protein 5 isoform 1 (Homo sapiens)	252.15	43.38	24.6	4.83	Cell structure and mobility. Found in hepatocellular carcinoma and hematopoietic tumorigenesis; involved in transformation, proliferation, invasion, and metastasis in anaplastic large-cell lymphoma	[30,70]

Note: List of protein identification. Spot: number of spots selected in accordance with 2-DE gel.

## Data Availability

The data presented in this study are available on request from the corresponding author.
